# Can we correlate pelvic floor dysfunction severity on MR defecography with patient-reported symptom severity?

**DOI:** 10.1007/s13304-017-0506-0

**Published:** 2017-12-19

**Authors:** Lisa Ramage, Panagiotis Georgiou, Shengyang Qiu, Paul McLean, Nasir Khan, Christos Kontnvounisios, Paris Tekkis, Emile Tan

**Affiliations:** 1 Department of Surgery and Cancer, Chelsea and Westminster Hospital, Imperial College London NHS Trust, Academic Surgery, 3rd Floor, Fulham Road, London, SW10 9NH UK; 2 Department of Colorectal Surgery, The Royal Marsden NHS Foundation Trust, London, UK; 3 Department of Radiology, Chelsea Westminster Hospital, London, UK; 4 Department of Radiology, The Royal Marsden NHS Foundation Trust, London, UK; 5 Department of Colorectal Surgery, Singapore General Hospital, Singapore, Republic of Singapore

**Keywords:** Patient-reported outcome measures, Magnetic-resonance defecography, Functional-bowel disorders

## Abstract

MR defecography (MRD) is an alternative to conventional defecography (CD) which allows for dynamic visualisation of the pelvic floor. The aim of this study was to assess whether MRI features indicative of pelvic floor dysfunction correlated with patient-reported symptom severity. MR proctograms were matched to a prospectively-maintained functional database. Univariate and multivariate analyses were performed using pre-treatment questionnaire responses to the Birmingham Bowel, Bladder and Urinary Symptom Questionnaire (BBUSQ), Wexner Incontinence Score (WIS), and modified Obstructed Defecation Symptom (ODS) Score. 302 MRI proctograms were performed between January 2012 and April 2015. 170 patients were included. Patients with a rectocele > 2 cm (*p* = 0.003; OR 5.756) or MRD features suggestive of puborectalis syndrome (*p* = 0.025; OR 8.602) were more likely to report a higher ODS score on multivariate analysis. Lack of rectal evacuation was negatively associated with an abnormal WIS (*p* = 0.007; OR 0.228). Age > 50 (*p* = 0.027, OR 2.204) and a history of pelvic floor surgery (*p* = 0.042, OR 0.359) were correlated with an abnormal BBUSQ incontinence score. Lack of rectal evacuation (*p* = 0.027, OR 3.602) was associated with an abnormal BBUSQ constipation score. Age > 50 (*p* = 0.07, OR 0.156) and the presence of rectoanal intussusception (*p* = 0.010, OR 0.138) were associated with an abnormal BBUSQ evacuation score. Whilst MRD is a useful tool in aiding multidisciplinary decision making, overall, it is poorly correlated with patient-reported symptom severity, and treatment decisions should not rest solely on results.

## Introduction

Magnetic-resonance defecography (MRD) was first introduced in 1993 [1] as a means of dynamic multi-compartmental visualisation of the pelvic floor. Its main advantages over fluoroscopic techniques, such as conventional defecography (CD), were the depiction of significantly higher anatomical detail of the pelvis achievable through T2-weighted images, and the ability to assess the movements of the anterior, middle, and posterior compartments of pelvis and its surrounding structures dynamically. The absence of ionising radiation enabled its use in a younger group of patients [2]. However, the utility of MRD over its X-ray counterpart needs further examination.

MRD has been shown to be useful in the diagnosis of pelvic floor dysfunction, particularly in the differential diagnosis of pelvic organ prolapse, evacuatory as well as continence disorders. It has utility in assessing the surrounding organs, in particular the uterus and vagina in women, and the dynamic interplay of the three pelvic compartments. MRD has also facilitated the development of three-dimensional interactive pelvic floor models [3, 4] which can be utilised to better understand the dynamic structural relationships of the pelvic floor. Several studies assessing predominantly posterior compartment dysfunction have found that added benefits of MR defecography over CD include the ability to identify enteroceles and the presence of levator ani hernias [5, 6].

Scoring systems for radiological diagnosis of severity of disease include the HMO classification [7] and grading systems based on the number of compartments affected [8]. These have concentrated on the presence/absence of enterocele, measurement of pelvic floor descent (M line), widening of the levator hiatus (H line), and the degree of cystocele and rectocele formation and middle compartment descent, using the pubococcygeal line (PCL) for Ref. [9].

Ultimately, clinicians use the findings of various imaging modalities in conjunction with patient-reported symptom severity to guide patient management. This is particularly important in functional disease, where an attempt to improve patient functional outcomes and quality of life is the indication for intervention rather than being in the context of malignancy or another life-threatening condition. Therefore, it is important to understand the relationship between anatomical severity on MRD and patient symptomology to guide treatment decisions.

The aim of this study was to assess the main radiological features examined in MRD and examine the extent to which their severity correlates with symptoms as reported by patients.

## Methods

All patients who had undergone MR defecography within a single tertiary referral centre for pelvic floor dysfunction between January 2012 and April 2015 were identified retrospectively from a prospectively-maintained database. This research database had full ethical approval. Three validated functional questionnaires were routinely given out during each outpatient clinic attendance to capture pre- and post-treatment data.

Patients with at least one of: Birmingham Bowel and Urinary Symptom Questionnaire (BBUSQ); Wexner (Cleveland Clinic) Incontinence Score; Obstructed Defecation Score (ODS), completed within 12 months of the MRD taking place and prior to any treatment intervention were identified and included within the study.

### Patient-reported outcome measures

The BBUSQ, a validated 22-point self-administered questionnaire for the assessment of urinary and bowel symptoms in females [10], has four domains: constipation, incontinence, evacuation, and urinary. Each domain is scored out from 0–100%, with the higher the score indicating more severe symptoms. As according to its validation criteria, an abnormal score for each domain was indicated as follows: constipation ≥ 64%, faecal incontinence ≥ 17%, evacuation ≥ 17%, and urinary ≥ 20%. With the exception of one question which refers to the presence of a bulge in the vagina, all questions were applicable to both males and females presenting with pelvic floor dysfunction, therefore, this questionnaire was used in both males and females, with the aforementioned question removed in male patients and the evacuation score adjusted accordingly. This score was chosen as a self-reported functional comparative tool for this study as it represented both the anterior and middle compartments in its symptom assessment.

The Wexner incontinence score (WIS) was first published in 1993 [11] and has gained widespread acceptance. Its scoring system takes into consideration symptoms of solid or liquid faecal and flatal incontinence, in addition to the need to use pads/medication to aid continence and the need to alter lifestyle due to problems with incontinence. A WIS of ten or more in this study was considered to be indicative of significant symptoms of faecal incontinence.

The obstructive defecation symptom score (ODS score) used for the purposes of this study was adapted from the original score developed by Longo et al. [12] and comprised of five questions, each scoring a maximum of four points relating to the presence of: (1) constipation; (2) straining; (3) incomplete evacuation of stool; (4) the need to interdigitate the rectum or vagina or use perineal support to initiate defecation; and (5) laxative or enema use. A score of 8 or above out of 20 was considered to be abnormal.

For each of the four BBUSQ domains, the Wexner Incontinence Score and the ODS score, patients were marked as either having a normal or abnormal score as according to the above criteria.

### MRI proctogram protocol

The Siemans Avanto 1.5 Tesla MRI scanner was used for all examinations. Patients were not administered bowel preparation or enemas. 30–50 ml of ultrasonic gel was inserted into the vagina in females, and 50–100 ml was inserted into the rectum. Patients were also advised to have a full bladder prior to the examination. Patients were positioned in the supine position within the MRI scanner. Initial T2-weighted turbo spin echo (TSE) images were acquired in 3 mm slices through the pelvis in sagittal, coronal, and axial directions.

An initial sagittal image centred on the anal sphincter complex was taken. Next, Trufisp (TE: 1.69 s; TR: 4.26 s) 5 mm thickness images were rapidly acquired in 25-image sequences. A single dynamic run was acquired, whilst the patient was instructed to contract their pelvic floor. Following this, the patient was instructed to empty their rectum whilst on the scanner. 2–3 dynamic sequence runs were taken during this evacuation phase.

### MRI interpretation

All MRIs were interpreted on two separate occasions by two reviewers (NK & LR). They were blinded to clinical findings. In instances where there was disagreement in scan interpretation, a third opinion was sought from a consultant colorectal surgeon with a specialist interest in pelvic floor pathology and experience in interpretation of MR proctograms (ET).

In cases where the patient had completely failed to follow instructions during the MRD, then they were excluded. This was defined by failure to expel any contrast whatsoever from either the vagina in females or rectum and no evidence of dynamic pelvic floor movement on the strain/evacuatory phase.

The pubococcygeal line (PCL) (from inferior border of pubic symphysis to tip of coccyx) was used as a fixed reference point for all measurements taken during rest and maximal pelvic floor strain as per standard reporting guidelines for MRD [13]. For each case, each MRI variable was coded into a binary score, indicating the presence or absence of that abnormality. In addition, where specific measurement parameters had been predefined in the literature, then the data were also separately coded to indicate ‘no/minor abnormality’ or ‘moderate/severe abnormality’.

**Fig. 1:**
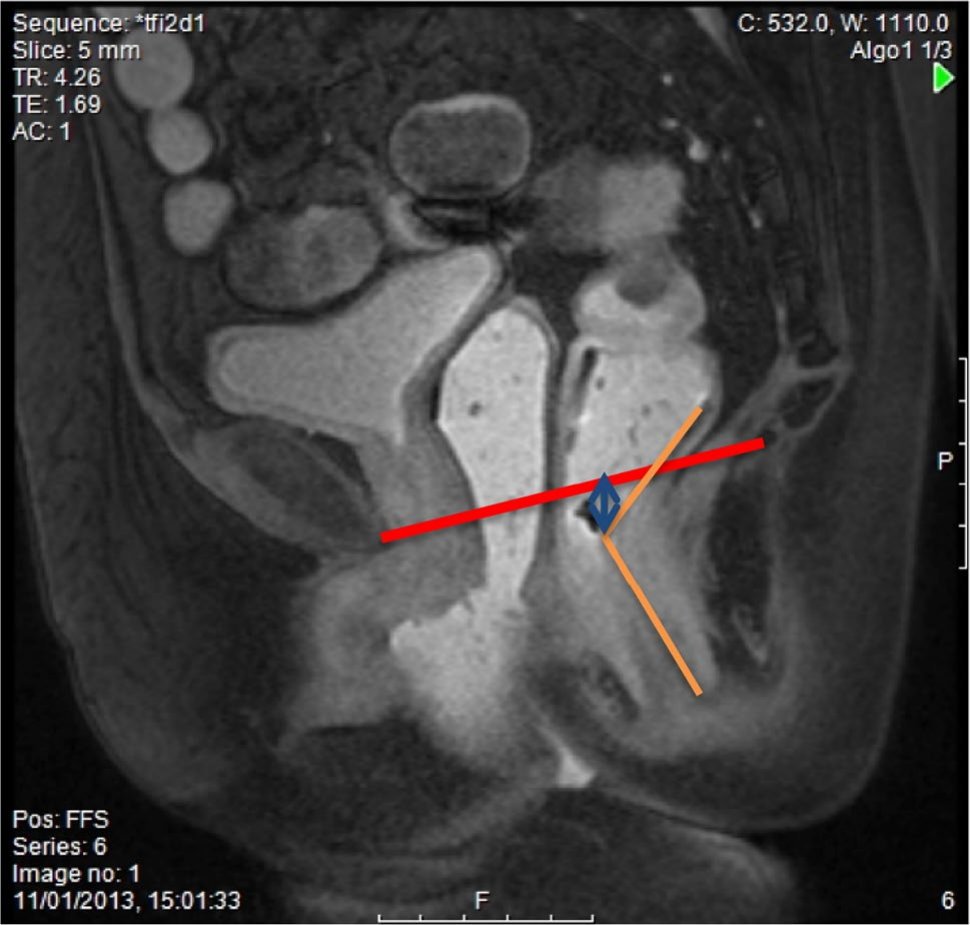
Pubococcygeal line (PCL) (red) is a fixed point of reference in MR defecography interpretation, drawn between the inferior border of the pubic symphysis and the coccyx. On the static (rest) image, the anorectal angle at rest (orange) was noted, in addition to the position of the anorectal junction (blue arrow) (colour figure online)

MRI variables were as follows:

On initial static image (Fig. 1):

1. Evidence of hysterectomy.
2. M line at rest (perpendicular distance from PCL to anorectal junction).
3. Anorectal angle.

**Fig. 2:**
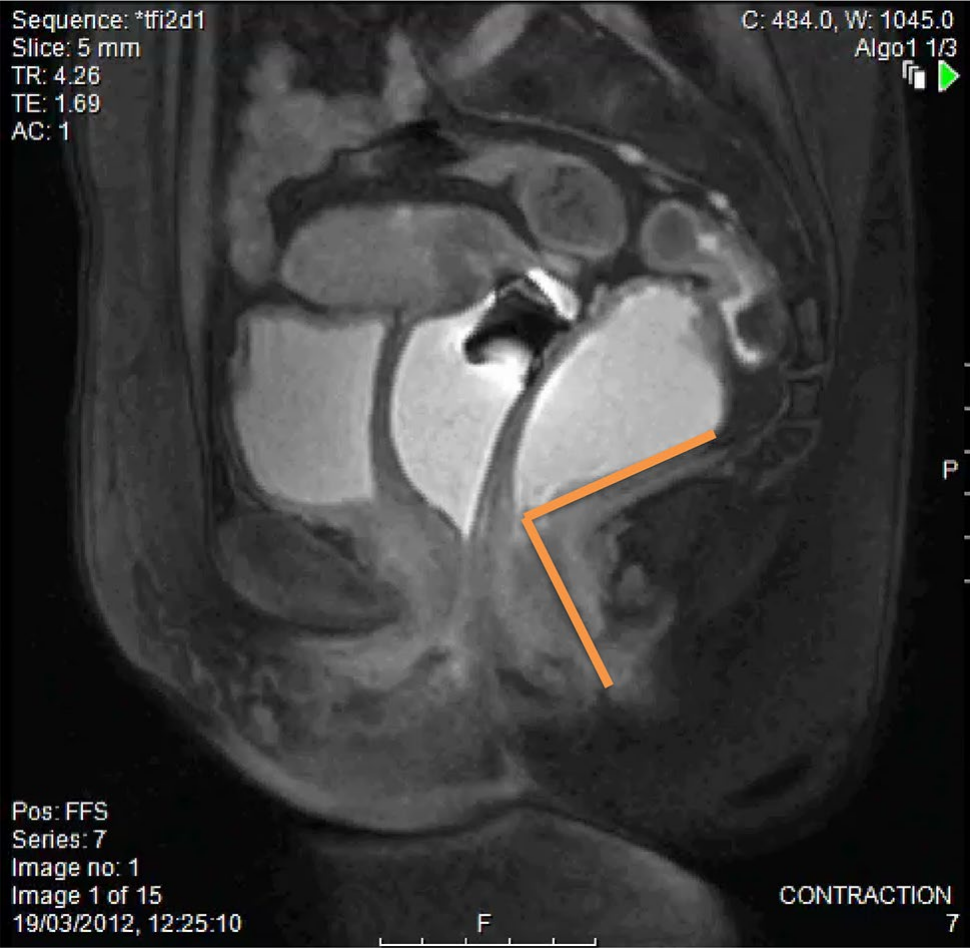
Patient is asked to contract and elevate the pelvic floor. The anorectal angle (ARA), (orange) should become more acute. A change of ten degrees or less was considered abnormal (colour figure online)

On pelvic floor contraction (Fig. 2):

1. Evidence of pelvic floor elevation (defined as a decrease in the anorectal angle by at least 15°).

**Fig. 3:**
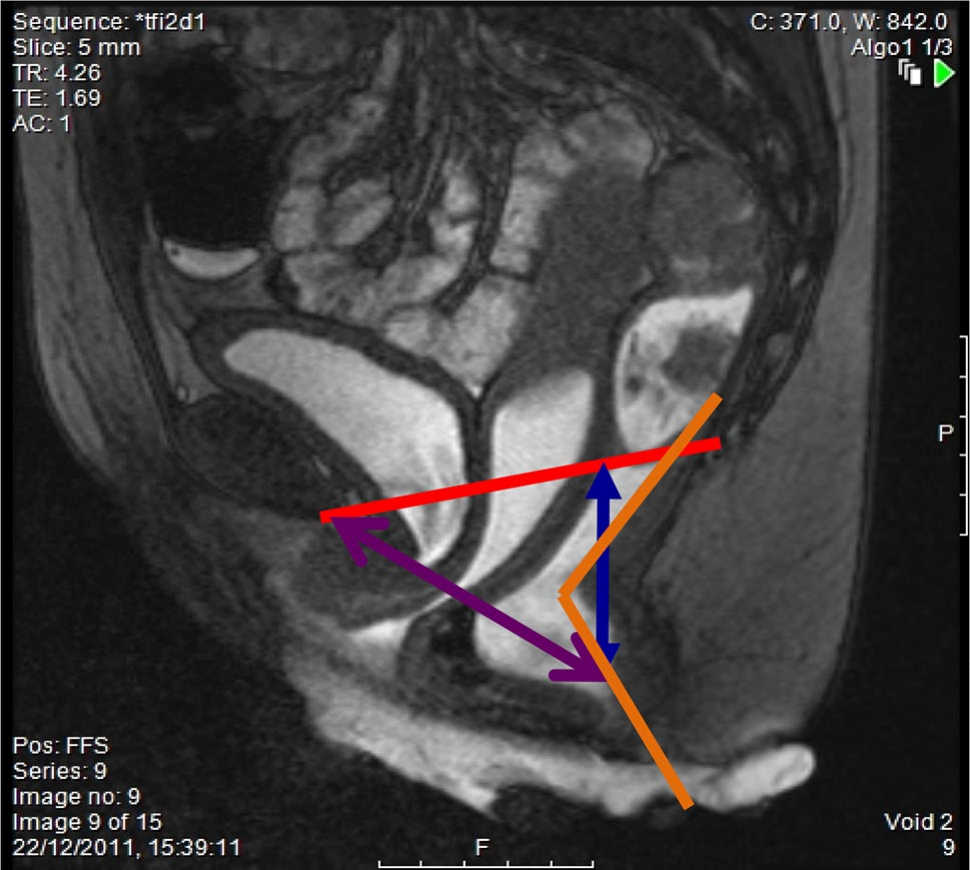
On strain views, the PCL (red) is again the reference point. The M line (blue arrow) indicates the degree of descent of the anorectal junction on attempted defecation. The anorectal angle (orange) should increase by 15°–20°. An abnormal H line (purple arrow) indicates excessive hiatal widening and pelvic floor laxity (colour figure online)

On maximal pelvic strain (Fig. 3):

1. M line: indicating pelvic floor descent. Descent of greater than 2 cm was considered abnormal).
2. H line (distance from inferior border of pubic symphysis to anorectal junction): indicating hiatal widening.
3. Anorectal angle (an increase in the angle of more than 15° was considered to be normal).
4. Presence/degree of cystocele (defined as any prolapse of the bladder more than 1 cm below the PCL).
5. Presence of urethral hypermobility (horizontalisation of the urethra on straining by > 30°).
6. Presence/degree of middle compartment descent (defined as any prolapse of the vaginal vault or posterior cervical fornix below the PCL).
7. Presence/degree of rectocele (defined as a bulge in the anterior rectal wall).
8. Presence/degree of enterocele (defined as the presence of small bowel below the PCL).
9. Presence of mucosal prolapse/rectoanal intussusception/rectal prolapse.
10. Degree of emptying of rectum (less than 80% emptying of the gel was considered abnormal).
11. Evidence of paradoxical puborectalis syndrome (features included thickening of the internal anal sphincter, the inability to empty the rectum, and failure of the anorectal angle to increase on attempted defecation, or the anorectal angle to become more acute on evacuation attempts).

Patient age over 50 and a history of prior pelvic floor surgery were also included as covariates in analysis.

### Statistical analysis

SPSS was used for all data analysis. Pearson Chi square was used to compare distributions across sex and age for each variable. For each domain, binomial logistic regression analysis was performed through univariate analysis of MRI covariate for each BBUSQ domain, Wexner Score and ODS score. Factors with a *p* < 0.2 on univariate analysis were included in multivariate analysis. *p* < 0.05 was considered to be statistically significant.

## Results

A total of 302 MRD were performed over a 39-month period between January 2012 and April 2015. Of these, 191 (63.25%) had documented functional data in the form of at least one of: BBUSQ, Wexner Incontinence Score, or the ODS score which was undertaken within 12 months of the patient undergoing MRD and prior to any further treatment intervention. 21 patients were excluded: 8—no/little pelvic floor movement during evacuatory phase; 1—image improperly centred, therefore, difficult to interpret; 3 score > 1 year; 1—MRD scan performed in context of fistula rather than PF dysfunction; 3—insufficient data entered on database; and 5—functional data > 1 year.

Therefore, a total of 170 patients were included in this study. Median age was 55 years (range 17–105). 57.1% of patients were older than 50 years. 151 (88.8%) were females. 30 (19.9%) females had undergone prior hysterectomy. 25 patients (14.7%) had previously undergone other form of pelvic floor surgery (10 stapled transanal resection of the rectum, STARR; 4 anterior resection; 1 total colectomy and J-pouch; 3 ventral mesh rectopexy, VMR; 2 rectocele repair, and 5 anterior/posterior vaginal repair).

107/170 (62.9%) patients demonstrated little to no rectal evacuation during the examination. Only 31 (18.2%) patients were able to successfully expel > 80% of the rectal contents during the examination.

### BBUSQ score: constipation domain

The BBUSQ constipation domain score data were available for 157 patients (92.3%). 47 patients (29.9%) had an abnormal score, i.e., ≥ 64%. 33.1% of females versus 10.5% of males had an abnormal score (*p* = 0.045). 37.7% of patients under the age of 50 versus 23.9% of those over 50 years of age had an abnormal score (*p* = 0.061).

### Univariate analysis (Table 1)

On univariate analysis, only the inability to evacuate 80% of rectal contrast was associated with an abnormal constipation score (*p* = 0.039, OR 3.235; 95% CI 1.059–9.881). Age > 50 (*p* = 0.062, OR 0.518; 95% CI 0.260–1.034) and female sex (*p* = 0.062, OR 4.204; 95% CI 0.931–18.978) approached, but did not reach statistical significance.

**Table 1:** BBUSQ: univariate analysis (only MRI variables with *p* < 0.2 shown)

Covariate	Odds ratio	95% Confidence limit lower upper		*p* value
Constipation				
Age > 50	0.52	0.26	1.03	0.062
Female Sex	4.20	0.93	18.98	0.062
Rectocele > 2 cm	1.57	0.79	3.12	0.193
<80% rectal evacuation	3.23	1.060	9.88	**0.039**
Incontinence				
Previous pelvic floor surgery	0.48	0.20	1.16	0.104
Abnormal PF elevation	1.69	0.81	3.53	0.166
M line > 2 cm	2.05	0.90	4.66	0.087
H line > 6 cm	2.41	1.20	4.83	**0.013**
<80% rectal evacuation	0.45	0.19	1.08	0.074
Age > 50	1.98	1.05	3.76	**0.036**
Evacuation				
Age > 50	0.16	0.05	0.58	**0.05**
Failure to increase anorectal angle	0.46	0.19	1.13	0.089
Presence of cystocele	2.22	0.86	5.74	0.100
Presence of urethral HM	3.40	1.18	9.75	**0.023**
Rectoanal intussusception	0.38	0.120	1.18	0.095
Urinary				
Prior hysterectomy	2.05	0.76	5.49	0.154
Abnormal elevation	2.27	1.01	5.10	**0.047**
M line > 4 cm	1.65	0.85	3.20	0.135
H line > 6 cm	2.46	1.21	4.99	**0.013**
Presence of cystocele	2.19	1.12	4.29	**0.022**
Presence of cystocele > 3 cm	2.38	0.84	6.76	0.104
Presence of rectocele > 2 cm	1.95	1.00	3.80	**0.049**
Urethral hypermobility	1.95	0.99	3.86	0.054
Female sex	0.59	0.19	1.38	0.189
Previous pelvic floor surgery	0.50	0.21	1.20	0.121

Statistically significant *p* values are in bold (*p* < 0.05)

### Multivariate analysis (Table 2)

Similarly, on multivariate analysis, the inability to evacuate of more than 20% of the rectal contents was the only statistically significant factor in the prediction of an abnormal score (*p* = 0.027, OR 3.602; 95% CI 1.159–11.197).

**Table 2:** Multivariate analysis BBUSQ

Covariate	Odds ratio	95% Confidence limit		*p* value
		Lower	Upper	
Constipation				
<80% rectal evacuation	3.60	1.16	11.20	**0.027**
Age > 50	0.57	0.28	1.18	0.130
Female sex	0.24	0.05	1.15	0.074
Rectocele > 2 cm	1.24	0.59	2.64	0.572
Incontinence				
Age > 50	2.20	1.09	4.45	**0.027**
Previous PF surgery	0.36	0.13	0.96	**0.042**
Abnormal elevation	1.35	0.61	2.98	0.458
M line > 2 cm	0.86	0.24	3.08	0.822
H line > 6 cm	2.64	0.87	8.01	0.087
<80% rectal evacuation	0.51	0.19	1.38	0.188
Evacuation				
Age > 50	0.16	0.04	0.60	**0.007**
Abnormal change in Anorectal angle	0.79	0.26	2.41	0.681
Presence of cystocele	2.42	0.43	13.48	0.314
Presence of urethral hypermobility	2.45	0.41	14.66	0.328
Presence of rectoanal intussusception	0.14	0.03	0.62	**0.010**
Urinary				
Previous pelvic floor surgery	0.27	0.09	0.86	**0.026**
Presence of urethral hypermobility	1.25	0.44	3.59	0.672
H line > 6 cm	2.27	0.67	7.67	0.188
Abnormal elevation	2.46	0.92	6.60	0.073
Prior hysterectomy	1.86	0.62	5.60	0.271
M line > 4 cm	0.57	0.19	1.70	0.311
Presence of rectocele > 2 cm	1.99	0.71	5.62	0.191
Cystocele > 3 cm	2.04	0.53	7.84	0.300

Statistically significant *p* values are in bold (*p* < 0.05)

### BBUSQ score: faecal incontinence score

164 (96.5%) patients had a completed dataset to allow for calculation of the incontinence domain score. 61.0% of patients had an abnormal score, i.e., ≥ 17%. 51.4% of patients under 50 versus 67.7% of those over 50 years of age had an abnormal score (*p* = 0.035). 61.4% of females and 57.9% of males had an abnormal score (*p* = 0.770).

### Univariate analysis (Table 1)

On univariate analysis, the presence of an abnormal H line of more than 6 cm (*p* = 0.013, OR 2.411; 95% CI 1.23–4.834) and age of more than 50 (*p* = 0.036, OR 1.983; 95% CI 1.047–3.759) were the only factors of statistical significance. Descent of the anorectal junction of more than 2 cm below the pubococcygeal line (M line) (*p* = 0.087, OR 2.049; 95% CI 0.901–4.656) and lack of rectal evacuation (*p* = 0.074, OR 0.452; 95% CI 0.189–1.081) approached, but did not reach statistical significance.

### Multivariate analysis (Table 2)

On multivariate analysis, patients over 50 were 2.2 times more likely to have an abnormal faecal incontinence score on the BBUSQ (*p* = 0.027, OR 2.204 95% CI 1.092–4.447).

A history of the previous pelvic floor surgery was negatively associated with an abnormal faecal incontinence score (*p* = 0.042, OR 0.359; 95% CI 0.134–0.962).

### BBUSQ score: evacuation domain

160 patients (94.1%) had sufficient data to calculate the evacuation domain score. 85.7% of patients had an abnormal evacuation score (i.e., ≥ 17%). 85.9% females versus 83.3% in males (*p* = 0.769). 95.6% of patients under 50 versus 78.0% over 50 reported an abnormal score (*p* = 0.002).

### Univariate analysis (Table 1)

On univariate analysis, age of more than 50 (*p* = 0.05, OR 0.164; 95% CI 0.047–0.577; 0.187–1.127) and the presence of urethral hypermobility (*p* = 0.023, OR 3.40, 95% CI 1.186–9.746) were found to be statistically significant for the prediction of an abnormal score.

Failure to change the anorectal angle by 15° or more (*p* = 0.089, OR 0.459, 95% CI) and the presence of rectoanal intussusception or rectal prolapse (*p* = 0.095, OR 0.377; 95% CI 0.120–1.185) showed a trend towards statistical significance.

### Multivariate analysis (Table 2)

On multivariate analysis, age > 50 (*p* = 0.07, OR 0.156; 95% CI 0.041–0.600) and the presence of rectoanal intussusception or rectal prolapse (*p* = 0.010, OR 0.138; 95% CI 0.031–0.623) were the only factors found to be predictive of an abnormal evacuation score.

### BBUSQ score: urinary domain

159 (93.5%) of patients supplied sufficient data to calculate the urinary domain of the BBUSQ. 64.1% indicated abnormal score of > 20%. 58.8% of patients under 50 versus 68.1% of those over 50 reported an abnormal score (*p* = 0.226). 66.0% of females versus 50% of males reported an abnormal urinary domain score (*p* = 0.184).

### Univariate analysis (Table 1)

On univariate analysis, abnormal pelvic floor contraction and elevation (*p* = 0.047; OR 2.050 95% CI 0.765–5.494), hiatal widening > 6 cm (*p* = 0.013; OR 2.460 95% CI 1.211–4.998), presence of a cystocele (*p* = 0.022; OR 2.191 95% CI 1.120–4.289), and presence of a rectocele > 2 cm (*p* = 0.049; OR 1.950 95% CI 1.001–3.798) were associated with an abnormal domain score.

### Multivariate analysis (Table 2)

On multivariate analysis, the only factor found to be statistically correlated with an abnormal score was a history of prior pelvic floor surgery; patients were 0.27 times as likely to have urinary symptoms following prior pelvic floor surgery (*p* = 0.026; OR 0.271 (95% CI 0.086–0.855).

## ODS score

The ODS score was completed by 136 patients (80%) in total. 77 patients (66%) indicated an ODS score of 8 or more, which was taken to indicate clinically significant symptoms. 72.7% of females versus 46.7% of males had a score > 8 (*p* = 0.038). 65.3 of those over 50 versus 75% of those aged under 50 had a high ODS score (*p* = 0.225).

### Univariate analysis (Table 3)

The presence of a rectocele > 2 cm was significantly correlated with an abnormal ODS score (*p* = 0.001; OR 4.007 95% CI 1.763–9.109), as was female sex (*p* = 0.045; OR 3.048 95% CI 1.024–9.068). The presence of a cystocele (*p* = 0.058; OR 2.109 95% CI 0.975–4.559) and MRI evidence suggestive of puborectalis syndrome (*p* = 0.096; OR 3.656 95% CI 0.796–16.788) were approaching statistical significance.

**Table 3:** ODS score (univariate)

Covariate	Odds ratio	95% Confidence interval		*p* value
		Lower	Upper	
Failure to increase anorectal angle	1.84	0.80	4.20	0.148
M line > 4 cm	1.89	0.88	4.04	0.101
H line > 8 cm	1.89	0.81	4.43	0.142
Presence of cystocele	2.11	0.97	4.56	0.058
Rectocele > 2 cm	4.01	1.76	9.11	**0.001**
Puborectalis syndrome	3.66	0.80	16.79	0.096
Female sex	3.05	1.02	9.07	0.045
Previous pelvic floor surgery	2.76	0.76	10.00	0.122

Only variables with a *p* < 0.2 shown

Statistically significant *p* value is in bold (*p* < 0.05)

### Multivariate analysis: Table 4

Patients with a rectocele of 2 cm or more in size were significantly more likely to have an abnormal ODS score (*p* = 0.003; OR 5.756 95% CI 1.829–18.112). Similarly, those who had features suggestive of puborectalis syndrome were significantly more likely to report a higher ODS score (*p* = 0.025; OR 8.602 95% CI 1.318–56.158). No other factors on univariate analysis approached statistical significance on multivariate analysis.

**Table 4:** ODS score—multivariate analysis

Covariate	Odds ratio	95% Confidence interval		*p* value
		Lower	Upper	
Rectocele > 2 cm	5.76	1.83	18.11	**0.003**
Failure to increase anorectal angle	2.03	0.67	6.17	0.211
M line > 4 cm	0.63	0.17	2.33	0.492
H line > 8 cm	0.93	0.24	3.66	0.916
Puborectalis syndrome	8.60	1.32	56.16	**0.025**
Female sex	2.76	0.62	12.36	0.185
Previous pelvic floor surgery	2.42	0.56	10.55	0.239
Presence of cystocele	2.23	0.781	6.37	0.134

Statistically significant *p* values are in bold (*p* < 0.05)

### Multivariate analysis (Table 4)

On multivariate analysis, patients with a rectocele of 2 cm or more in size were significantly more likely to have an abnormal ODS score (*p* = 0.003; OR 5.756 95% CI 1.829–18.112). Those who had features suggestive of puborectalis syndrome were more likely to report a higher ODS score (*p* = 0.025; OR 8.602 95% CI 1.318–56.158). No other factors on univariate analysis approached statistical significance on multivariate analysis.

## Wexner incontinence score

The Wexner incontinence score was completed in 153 patients (90.0%). Scores were ≥ 10 in 30.1% of patients. 24.2% of those under 50 versus 34.4% of those over 50 (*p* = 0.176) had a WIS score ≥ 10. 30.4% of females versus 27.8% of males had a high WIS (*p* = 0.822).

### Univariate analysis (Table 5)

On univariate analysis, prior hysterectomy (*p* = 0.006; OR 3.446 95% CI 1.432–8.295), presence of enterocele (*p* = 0.003; OR 5.667, 95% CI 1.815–17.697), and inability to evacuate 80% of rectal contents during MR proctogram (*p* = 0.001; OR 0.237 95% CI 0.101–0.556) were found to have statistical correlation with an abnormal Wexner incontinence score.

**Table 5:** Wexner incontinence score—univariate analysis

Covariate	Odds ratio	95% Confidence interval		*p* value
		Lower	Upper	
Hysterectomy	3.45	1.43	8.29	**0.006**
Elevation	2.03	0.94	4.38	0.073
Enterocele	5.67	1.81	17.70	**0.003**
Mucosal binary	1.86	0.91	3.81	0.090
Evacuation < 80%	0.24	0.10	0.56	**0.001**
Age > 50	1.64	0.98	3.40	0.178

Only variables with a *p* < 0.2 shown

Statistically significant *p* values are in bold (*p* < 0.05)

### Multivariate analysis (Table 6)

Multivariate analysis demonstrated significant correlation only with lack of rectal evacuation and the Wexner Incontinence Score (*p* = 0.007; OR 0.228 95% CI 0.079–0.664).

**Table 6:** Wexner incontinence score—multivariate analysis

Covariate	Odds Ratio	95% Confidence interval		*p* value
		Lower	Upper	
Age > 50	1.85	0.70	4.90	0.215
Hysterectomy	1.57	0.51	4.81	0.425
Evacuation < 80%	0.23	0.08	0.66	**0.007**
Mucosal intussusception	0.88	0.33	2.39	0.808
Presence of enterocele	2.95	0.72	12.11	0.133
Abnormal pelvic floor elevation	1.41	0.54	3.67	0.480

Statistically significant *p* value is in bold (*p* < 0.05)

## Discussion

The present study aimed to assess whether features suggestive of pelvic floor dysfunction as seen on MR defecography had any correlation with patient-reported severity of symptoms. Overall, with the exception of the findings reported above, there was little to no correlation between patient’s symptoms and MRI findings. Patient factors such as age over 50 and a history of prior pelvic floor surgery appeared to be more reliably linked to MRI findings.

The majority of MRI measurements seemed to have little to no bearing on symptom severity on regressional multivariate analysis. This seems surprising with factors such as the presence of an abnormal M line, which is indicative of perineal descent; however, only 28/170 (16.5%) patients had descent of the anorectal junction of less than 2 cm on strain, and 80 patients (47.1%) had moderate/severe anorectal descent (> 4 cm); therefore, this may have confounded the results. All patients included in this paper had undergone MRD as an investigation for the presence of symptoms. Therefore, it may be reasonable to conclude that anorectal junctional descent is a common and fairly early sign of pelvic floor weakness, and may commonly be present prior to the presence of significant symptoms.

A few studies to date have considered the relationship between patient-reported symptom severity and MR defecography. Broekhuis et al. [14] in 2009 undertook a study, where they analysed POP-Q results and MR findings against symptoms of pelvic organ prolapse in 69 patients. They found no predictive value of MRD in symptom severity; the only factor that did correlate with patient symptom severity was being able to see or feel a bulge in the vagina. Similarly, Lakeman et al. [15] attempted to correlate MRI with both patient-reported symptoms and clinical assessment in three groups of ten patients: 1: symptomatic patients with at least stage 2 pelvic organ prolapse (POP); 2: mild symptoms with stage 1 POP; and 3: asymptomatic nulliparous women. They compared three functional questionnaires: Urogenital Distress inventory, Defecation Distress Inventory, and the Incontinence Impact Questionnaire and clinical examination findings to MRI findings. None of the groups showed any positive correlation between MRI quantification of organ prolapse severity and questionnaire scores.

Piloni et al. [8] attempted evaluation of the use of a radiological classification system of obstructive defecation syndrome (ODS) to guide patient management. MR findings were classified into grades 1–5, with grade 1 showing functional disturbance only, grade 2 showing minor anatomical defects (e.g., rectocele < 2 cm, 1st degree intussusception), grade 3 showing major posterior compartment defects (e.g., rectocele > 2 cm or trapping, rectal floor descent below PCL > 5 cm), grade 4 showing major defects in all three compartments, and grade 5 showing surgical failure (e.g., ODS recurrence after STARR/stapled haemorrhoidectomy). Treatment recommendations were made based on the above scoring system, with grades 1–2 deemed suitable for pelvic floor rehabilitation, and surgical intervention for grades 3–5, with MDT input for recurrent complex cases. In addition, they attempted to correlate ODS (79) score and MR findings, and similarly failed to identify a robust relationship between the two. Hubner et al. [16] found in their series of 16 patients who underwent MR defecography pre- and post-corrective surgery for rectocele that there was a strong association between the clinical sensation of incomplete rectal evacuation and stool trapping on MR defecography; however, patient-reported symptoms did not correlate with preoperative rectocele size, nor with postoperative morphology of the corrected rectocele.

The reasons for the lack of significant correlation are likely multifactorial. The patient positioning within the MRI scanner is supine and, therefore, is not physiological. The validity of obtained results when the patient is lying supine as opposed to sitting in a normal position for defecation has been the subject of the previous debate within the literature. Bertschinger et al. [17] compared 32 patients who initially underwent the procedure in a supine position before repeating in a sitting procedure in an open magnet unit. That study showed that in both positions, evaluation of all three compartments was feasible. Rectal descent and anterior rectoceles were detected in both supine and sitting positions. However, small abnormalities such as small rectal descent, small anterior rectocele, and small vaginal vault descents were less well detected in supine position. The study’s conclusion was that although most abnormalities could be detected in a supine position, sitting MR was superior in detecting pelvic floor laxity, enteroceles, and anterior rectoceles. Fielding et al. [18] demonstrated that pelvic floor laxity in relation with urinary stress incontinence could be detected by either position; however, changes were more pronounced in a sitting position. Therefore, we could postulate that the supine positioning of the patient within the MRI may had led to under-representation of the presence or degree of clinically significant pelvic floor abnormalities. This makes the measurements of H and M lines even more vital, since they serve as a guide to deformatory strains on the rectum and the adjacent organs. The loss of descent due to gravity could be compensated for by informing patients of the need to really engage with the examination and strain as maximally as possible, since abdominopelvic strain is the predominant action bringing about tissue deformation in the pelvis. One should, therefore, be able to produce the required changes similar to a conventional sitting examination provided enough time is given, and instructions are clear and understood by the patient.

In our series, the defecatory phase of the MRD was performed with very variable success, with 70.9% of patients not demonstrating any evacuation of the rectum whatsoever. Pilkington et al. [19] in their comparative study of MRD versus barium proctography in 71 patients found an under-reporting of abnormalities with MRD in particular when there was lack of rectal evacuation with only 2% able to fully empty the rectum during MRD.

The importance of the defecatory phase was highlighted by Flusberg et al. [2] using MR defecographic examinations from 85 patients. An assigned score was given for each positive finding based on its severity, and scores were assessed for the rest, squeeze, strain, and defecation phases. The average defecation phase score was found to be significantly higher than the average scores in any other phase. There were significantly more rectoceles, enteroceles, and intussusceptions identified during the defecation phase. In addition, the degree of bladder, uterovaginal, and anorectal descent was significantly more marked. The reasons as to why patients were unable to empty their rectums were unclear in the majority of cases, and therefore, factors such as patient embarrassment, poor instruction by the radiographer, and too few attempts at defecation may have contributed to this. In addition, the use of a jelly-like medium as a contrast agent is in itself not a true representation of stool consistency, and therefore, this may in itself cause a misrepresentation of true evacuatory function.

## Limitations

The main limitation of this study is the lack of a control group of ‘healthy volunteers’. All patients who were studied had been referred due to the presence of symptoms, and therefore, this may explain both the high prevalence of symptoms and radiological abnormalities. Although not fully discussed in this paper, the authors did additionally analyse the data having moved the threshold of what was considered significant in terms of both PROMs and MRD parameters to assess whether correlations were more evident for more severe disease. Findings were not dissimilar to those already discussed; therefore, this was omitted from the manuscript. Moving forward, it would be our suggestion that any future work ensured a control group was used as a comparator. Another limitation worthy of note is the use of the BBUSQ, which has only been validated in female patients. The authors felt that this questionnaire gave a comprehensive assessment of both the anterior and posterior compartments, and omitted the one question not applicable to males and adjusted the scoring system accordingly. Despite the inclusion of two other scoring systems, which assessed bowel function, there was still little significance on multivariate analysis to allow the development of a scoring system to predict symptom severity and guide management. The exception to this was the ODS score which did show correlation with moderately-sized rectoceles and paradoxical puborectalis syndrome features on MRI, but not with other MRI factors such as descent.

In conclusion, whilst there were certain features on MRI defecography in addition to patient factors which are associated with abnormal scores on the three chosen scoring systems, there was no robust evidence of specific and consistent MRI features which could form part of a scoring system to allow the grading of symptom severity based on MRI findings alone. MR, however, remains a useful tool with advantages over conventional defecography in particular when there is a need to visualise the solid organs. We would recommend that MRD does have its role in particular with the assessment of complex multi-compartmental disease or those who have undergone prior pelvic floor surgery with distortion of normal anatomy.

Clinicians as a rule do not treat an image, but the patient; therefore, patient-reported symptom severity takes precedence when making treatment decisions. However, there is good evidence that if symptoms and radiology agree, then the combination of an anatomical and functional diagnosis together will likely increase the confidence of doctor and patient that an essentially functional treatment would be likely to succeed in positively affecting a patient’s symptoms.
